# Estimating the effect of multiple environmental stressors on coral bleaching and mortality

**DOI:** 10.1371/journal.pone.0175018

**Published:** 2017-05-04

**Authors:** Paul D. Welle, Mitchell J. Small, Scott C. Doney, Inês L. Azevedo

**Affiliations:** 1 Department of Engineering and Public Policy, Carnegie Mellon University, 5000 Forbes Ave. Pittsburgh, PA, United States of America; 2 Department of Civil and Environmental Engineering, Carnegie Mellon University, 5000 Forbes Ave., Pittsburgh, PA, United States of America; 3 Department of Marine Chemistry & Geochemistry, Woods Hole Oceanographic Institution, MS# 25, Woods Hole, MA, United States of America; Universidade de Aveiro, PORTUGAL

## Abstract

Coral cover has been declining in recent decades due to increased temperatures and environmental stressors. However, the extent to which different stressors contribute both individually and in concert to bleaching and mortality is still very uncertain. We develop and use a novel regression approach, using non-linear parametric models that control for unobserved time invariant effects to estimate the effects on coral bleaching and mortality due to temperature, solar radiation, depth, hurricanes and anthropogenic stressors using historical data from a large bleaching event in 2005 across the Caribbean. Two separate models are created, one to predict coral bleaching, and the other to predict near-term mortality. A large ensemble of supporting data is assembled to control for omitted variable bias and improve fit, and a significant improvement in fit is observed from univariate linear regression based on temperature alone. The results suggest that climate stressors (temperature and radiation) far outweighed direct anthropogenic stressors (using distance from shore and nearby human population density as a proxy for such stressors) in driving coral health outcomes during the 2005 event. Indeed, temperature was found to play a role ~4 times greater in both the bleaching and mortality response than population density across their observed ranges. The empirical models tested in this study have large advantages over ordinary-least squares–they offer unbiased estimates for censored data, correct for spatial correlation, and are capable of handling more complex relationships between dependent and independent variables. The models offer a framework for preparing for future warming events and climate change; guiding monitoring and attribution of other bleaching and mortality events regionally and around the globe; and informing adaptive management and conservation efforts.

## Introduction

Coral reefs provide important ecosystem services, such as sustaining fisheries, coastal protection, and social and cultural services such as recreation and tourism [1]. Worldwide, the goods and services provided by coral reefs are estimated to contribute nearly $30 billion US dollars annually [2]. Coral biodiversity and cover have been decreasing over the last three decades, and the trend is projected to accelerate as ocean temperatures continue to rise [3,4].

Environmental and climate change related stressors, such as temperature, solar radiation, and human interference are affecting these ecosystems, and the magnitude of such stressors will increase in the near future. The extent to which different stressors may contribute to bleaching and mortality of corals, however, is still largely unknown and uncertain.

A consensus is emerging that the warming of the oceans caused by anthropogenic climate change is leading to coral habitat destruction and will continue to do so into the future with likely very serious consequences [3–5]. Higher ocean temperatures have led to increased coral bleaching. As carbon dioxide concentrations increase, ocean pH can be expected to decline, retarding the corals’ ability to produce their calcium carbonate exoskeleton [6] and affecting larval settlement and recruitment [7]. Temperature and pH have been seen to act in a synergistically destructive manner, each amplifying the others’ effect to cause increased bleaching and mortality [8,9]. Solar radiation arriving at corals is also likely to change with the climate, due to its sensitivity to aerosol and cloud concentrations [10].

Undoubtedly, the most studied driver of habitat loss has been anomalously high temperatures. Elevated temperatures have consistently been seen to correlate with coral bleaching and death [11–15]. Results carried out from controlled laboratory experiments have confirmed these findings [8,16], and future increasing temperatures can be seen to be a major threat to corals worldwide. However, studies focusing on site condition, rather than controlled laboratory experiments, are still lacking.

Coral bleaching, defined as the loss of coral pigmentation from algal symbionts, was first observed nearly a century ago. It has recently become an issue of much concern as sea temperatures rise and mass bleaching episodes become more common [17]. Bleaching leaves corals less able to generate energy from algal photosynthesis, more vulnerable to diseases, and less able to engage in spawning and reproduction [18]. Temperature anomaly has been widely identified as the chief driver in this phenomenon. However, the marginal sensitivity of coral health to temperature changes is still fairly uncertain. In addition, there are other contributors that are likely to intensify in the near future due to shifting climate and increased human interference. Variations in solar radiation, sedimentation, and abundance of herbivorous fish have been seen to affect coral health and can be linked to human action [19].

Solar radiation, either in the form of ultraviolet (UV) or photosynthetically active radiation (PAR), has been seen to cause damage to exposed corals, in some cases bleaching a number of corals without presence of elevated temperatures [20,21]. Sudden increases in the amount of radiation received by corals have also been linked to bleaching [22]. In laboratory experiments, elevated radiation coupled with elevated temperature caused additional bleaching beyond that of temperature alone [8], further evidence of which was provided by Downs et al. [23] by using molecular biomarkers.

Humans often play a more direct role in harming coral ecosystems beyond affecting stressors driven by anthropogenic climate change. Improper diving and boating can lead to direct damage, which is becoming a significant problem in the Red Sea [24,25]. Overfishing, another anthropogenic stressor, reduces predation of the major coral competitor, macroalgae, and can cause increased likelihood of ecosystem phase shifts [26]. Proximity to land can leave corals at risk to fertilizer runoff and resultant eutrophication, adding more pressure to the ecosystem to shift to an algal dominated state [27]. Population density has been found to be a strong indicator of anthropogenic stress, and has been successfully used in the literature as a proxy variable for such stress [28,29]. Marine protected areas (MPAs) and land-use regulation are common techniques used to address these problems (e.g. [30–33]).

The depth at which corals are located has also been postulated to be important, as temperature and radiation attenuate with depth, but the direction of this effect is still fairly uncertain. For example, Mumby et al. [34] found an increased survival with deeper corals in French Polynesia, supporting results from earlier studies in Myrmidon Reef [35] and for a global analysis [36]. Deeper corals tend to be larger, slower growing, and more resistant to bleaching. The effect of depth is not always clear and will depend on several other factors, such as coral biology. For example, Marshall and Baird [37] found a significant spatial variation in bleaching, with some shallow sites reporting less bleaching than deeper ones. Williams and Bunkley-Williams [38] observe that “in the Florida Keys and Puerto Rico, bleaching started in the shallows and moved deeper; in Jamaica and St. Croix, the opposite was observed.” It is of great interest to develop an understanding of how all the above-identified stressors interact to impact coral health, so that this information can be combined with climate predictions to create a holistic understanding of the fate of coral reefs.

Thermal bleaching has been attributed to an overproduction of protons during the light reaction of the photochemical process [20]. Increased light and temperature accelerate the chemical processes associated with these reactions to the point where the dark reaction is unable to make use of all the energy and free radicals of oxygen are created, that are hypothesized to subsequently damage the host coral. Thermal bleaching is the expulsion of the algal symbionts, or zooxanthallae, presumably as protection against the various dangerous forms of oxygen [17]. In support of this theory, it has been observed empirically that free radicals are produced before bleaching occurs [39,40]. It also appears that bleaching may occur less in corals that have recently bleached, causing their levels of zooxanthallae to be lower than would otherwise be the case [21]. Additionally, the threshold for coral bleaching was found to be 1°C lower in winter when symbiont concentration is lowest [14].

Using tank experiments, Anthony et al. [8] sought to understand the interaction between temperature, light, and sedimentation, and found that increased turbidity protects corals from bleaching. While it is well-established in the literature that sedimentation is damaging to reefs [41], it is likely that in these tank experiments the turbid water shielded the corals from radiative damage or a radiation-temperature interaction effect [8]. The studies by Anthony et al. [8], Brown et al. [21], and others indicate that the biological pathway through which bleaching occurs indeed relies on excess solar radiation and temperature.

The National Oceanographic and Atmospheric Administration’s (NOAA’s) Coral Reef Watch (CRW) uses Degree Heating Weeks (DHW) to generate real-time warnings for areas at risk for bleaching. DHWs are computed by summing the number of degrees above maximum climatological monthly mean (MMM) for each week across the preceding twelve weeks (http://coralreefwatch.noaa.gov/), and therefore represent an estimate of how far above ‘typical’ values recent temperatures have strayed. Weeks with anomalies less than 1°C are considered to be non-anomalies and rounded to zero. Moderate bleaching is expected when DHW is larger than 4, and severe bleaching may occur with a DHW larger than 8. These data are made available in near real-time at a global scale.

A number of studies have attempted to explain the effect of temperature on coral bleaching using the DHW formulation and statistical models for regression analysis [42–46]. Existing analyses of coral bleaching typically involve one of two approaches: predicting the probability of being in a categorical stage of bleaching or resilient state (e.g., “high” or “low”) or predicting the fraction of corals bleached with an ordinary least squares (OLS) model. For example, McWilliams et al. [42] used the ReefBase (http://www.reefbase.org) data to predict bleaching severity and spatial extent using DHW, and found a log-linear increasing relationship, and Eakin et al. [43] performed a similar analysis with data exclusively from the 2005 bleaching event in the Caribbean, regressing bleaching on DHW using ordinary least squares (OLS).

Two major studies used metrics similar to degree heating weeks to assess what coral cover might look like in the future as temperatures rise. Hoegh-Guldberg [11] predicted the Degree Heating Months (DHM, similar to DHW) into the future, showing that DHM would rise beyond triple the levels currently experienced by as early as 2080. More importantly, DHM exceeding the worst values observed to date would become yearly events before the end of the century. Another study used similar metrics in the Caribbean to show that the DHM associated with the 2005 mass bleaching episode would reoccur roughly every other year by 2030–2050, depending on adaptation [5].

A few of these studies [42,43] use only temperature in predicting coral bleaching, but no study to date has attempted to predict coral mortality. Those studies that include additional predictor variables [44–46] find that variables such as PAR, depth, and wind speed lead to significant improvement in predicting coral health outcomes.

While a few of the above mentioned studies have attempted to determine the effect of temperature on coral bleaching using statistical and regression analyses [42–46], these studies have generally used an ordinary least-square (OLS) regression. There are several reasons why an OLS regression may not be an appropriate way to describe coral behavior in light of stressors, further elaborated upon in the methods. These studies have useful first order implications, but it is important to consider carefully whether temperature alone is sufficient for predicting future coral mortality. Theory and biological experimentation suggest that pH and radiation arriving at the corals will also be important in driving the photosynthesis reaction. If regressions of past temperature events are to be taken as predictive of future bleaching and mortality rates, it is important to consider the magnitude of the roles that each driver plays. Satellite radiation data available in the form of PAR or ultraviolet radiation (UVR) should be included as well as other environmental variables that might be relevant.

Yee, Santavy, and Barron [44] and Yee and Barron [45] extended the traditional analysis to include PAR taken from Moderate Resolution Imaging Spectroradiometer (MODIS) aboard NASA’s Terra satellite. The authors found that adding PAR (as well as other environmental variables and community taxonomic composition) increased *R*^*2*^ as well as resulted in lower corrected Aikaike Information Criterion (AIC_c_). These studies fit models to understand the probability of bleaching events. These early results, combined with data from the empirical work discussed above, indicate it may be crucial to incorporate a wider variety of environmental variables to understand coral health.

In this study, we use the observations of coral bleaching, mortality, and depth from a dataset compiled by Eakin et al. [43], which we describe in more detail in the data and methods section, as well as in the SI. The dataset includes 2945 measurements of coral health taken during the Caribbean summer of 2005 and is the most thorough measurement of basin-scale bleaching ever recorded. Major coral losses were observed in the Caribbean during the period for which measurements were made. During this period, record temperatures were set across the basin. In some reefs measured bleaching rates were as high as 95% [47]. We complement the dataset with weather data, specifically DHWs data from NOAA CRW and photosynthetically active radiation (PAR) from MODIS Aqua satellite, as well as distance from each coral reef to the nearest coastline, population density, wind speed and information about biogeographic regions. We provide more details regarding these data in the data and methods section and in the SI.

The work presented in this paper contributes to the literature by developing and using a novel regression approach, where the fraction of corals bleached and fraction of corals dead are estimated using a parametric non-linear model that controls for unobserved time invariant effects. Two separate models are created, one to predict coral bleaching, and the other to predict near-term mortality. A large ensemble of supporting data is assembled to control for omitted variable bias and improve fit, resulting in a significant improvement in predictive capacity.

## Materials and methods

### Data

We collect data from a variety of sources and combine them into a comprehensive statistical analysis of the coral death and bleaching process. The final dataset included observations on coral bleaching and mortality, temperature in the form of degree heating weeks, photosynthetically active radiation, and distance from shore. The following sections provide additional detail on these data.

#### Coral bleaching and mortality

**Fig 1:**
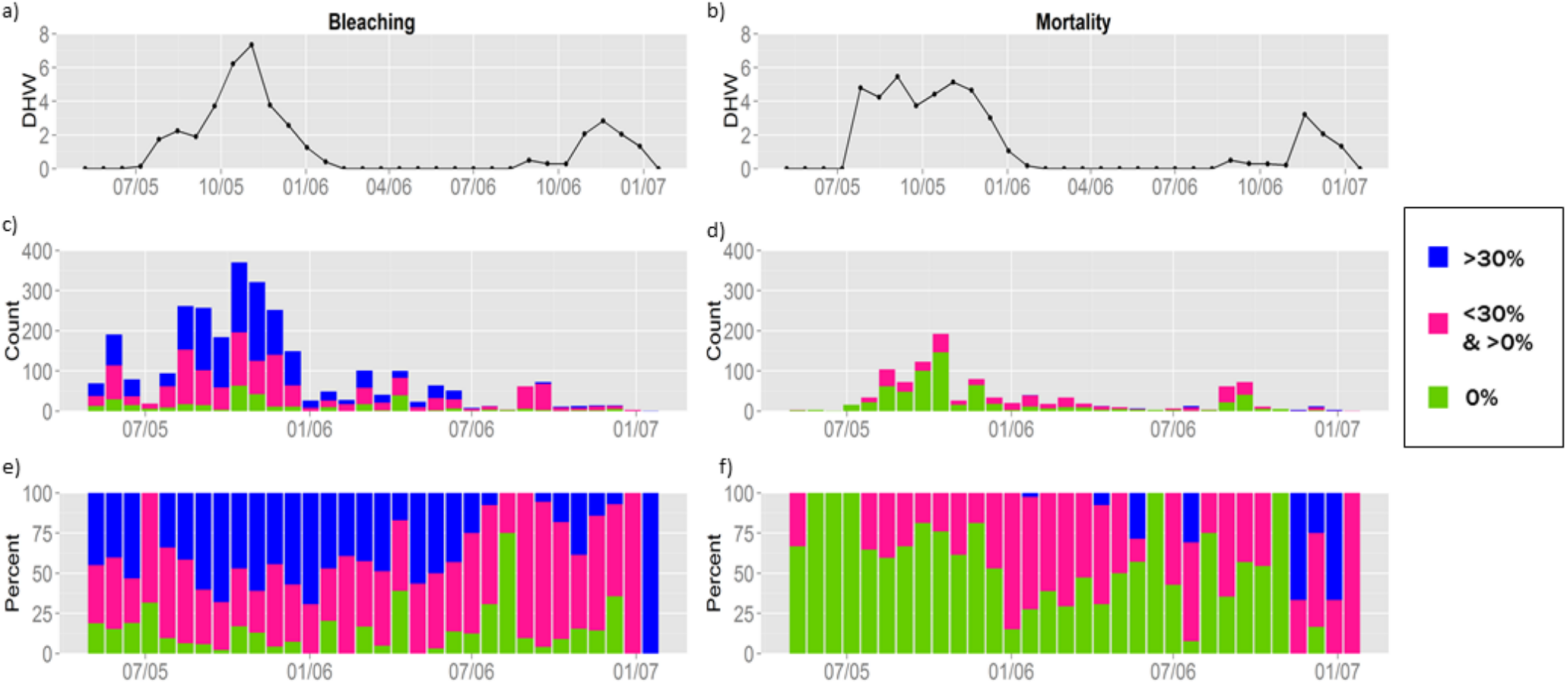
Temporal distribution of observations. Figures on the left column refer to bleaching, and those on the right refer to mortality. Fig 1A and 1B show the average DHW in the observed locations for the region of analysis between May 2005 and January 2007. Fig 1C and 1D show the number of observations, *x*, with 0%, 0% < *x* < 30%, or *x* >30% bleaching or mortality. Since the number of observed sites varies over time, in Fig 1E and 1F we show the share of observations in each distribution bin over time. The left and right panels for DHW, Fig 1A and 1B, differ because the observations available for mortality are a subset of those available for bleaching.

A dataset from Eakin et al. [43] is used to quantify the negative ecosystem effects observed in Caribbean reefs, with a total of 2,945 observations measured between May 2005 and January 2007. In SI, Section 1 (Additional Descriptive Statistics) we include more information on the data used and limitations associated with the dataset. Fig A in S1 File reports the spatial distribution of the observations, while the temporal distribution is reported in Fig 1. Fig 1A shows the average observed degree heating weeks (DHW) for the observations in that time period, and can be used to visualize the intensity of the temperature anomaly over time. The middle plots report the frequency of observations over time and are color coded to show the fraction of corals with different bleaching or mortality levels. Most observations were taken at the end of the summer of 2005, when temperatures were highest. The bottom graph illustrates the same information as the second graph with each bar normalized so trends in bleaching and mortality can be seen more clearly. Qualitatively, bleaching appears to be relatively stable while mortality increases with time.

The outcome measures of interest are coral bleaching and coral mortality. Each can be measured as a percentage of cover affected or colonies affected. *Cover bleached* represents the fraction of coral bleached in the observed area, while *colonies bleached* represent the fraction of bleached coral organisms relative to healthy organisms. Likewise, *cover dead* is the fraction dead per area and *colonies dead* is the fraction dead to living organisms. Eakin et al. [43] showed that these two measures, fractional area and fractional number of colonies, used independently give statistically indistinguishable results and uses this to justify selecting the average of the two as the dependent variable. In this study the same approach is followed and the average of the two measurement strategies is used, yielding one composite measure for bleaching and one for mortality.

It is not possible to assure that all bleaching and mortality are directly attributable to thermal stress (as opposed to, for example, disease or hurricanes). To address this concern, a measurement is included in the analysis only if it occurs after the first issuance of a thermal stress warning and before the 90^th^ day following the last no stress alert, as defined in the CRW Bleaching Alert System (http://coralreefwatch.noaa.gov). All bleaching identified in our data, therefore, occurred under the presence of thermal stress. As noted in Table 1, the spatial variation in temperature is still sufficient during the study period to yield a relatively wide range of DHW values, enabling its effects to be determined by the regression analysis.

**Table 1:** Summary statistics and variables’ description.

*Variable*	*Description*	*Units*	*Included in the mortality estimation model*	*Included in the bleaching estimation model*
Bleaching	Composite of cover bleached and colonies bleached	%		X
Mortality	Composite of cover dead and colonies dead	%	X	
Maximum^†^ DHW,	Temperature anomaly	°C	X	
Observed^‡^ DHW	Temperature anomaly	°C		X
Maximum PAR Anomaly	Radiation anomaly	Einstein / m^2^·day	X	X
Distance from Shore (DFS)	Shortest Euclidean path to shoreline	Kilometers		X
Population Density	Maximum population density within 50km of observation	1000 people per km^2^	X	X
Wind	Maximum wind speed before observation date	Meters / second	X	X
Depth	Depth of coral below surface	Meters	X	X
Depth×MaxPAR	Interaction terms between coral depth and maximum PAR anomaly	Meters x Einstein / m^2^·day	X	X
Depth×MaxDHW	Interaction terms between coral depth and maximum DHW	Meters x °C	X	
Depth×ObsDHW	Interaction terms between coral depth and observed DHW	Meters x °C		X

^†^Maximum stress is the most extreme values recorded since the beginning of the event (January, 2005).

^‡^Observed stresses are the values observed on the day of measurement.

Lastly, it should be noted that the sampling campaign did not follow a strict probabilistic approach, meaning that the corals measured may not perfectly represent the distribution of corals in the wider Caribbean. The sample itself is unique, however, in its size and extent and thus offers valuable insight into how changing climate and anthropogenic stress may be affecting coral health outcomes.

#### Temperature

Temperature is expressed in degree heating weeks, DHW. All temperature measures are calculated using data from NOAA's Coral Reef Watch (CRW). CRW maintains a historical database of observed and maximum DHW values, which were used in this analysis. All data are available at a 50km resolution. DHW are matched with observations based on time and location.

#### Photosynthetically active radiation (PAR)

We utilize satellite-derived estimates of the daily-averaged, photosynthetically available radiation (PAR) (400-700nm) just below the sea surface. PAR is a standard data product from NASA MODIS Aqua (http://modis.gsfc.nasa.gov/data/dataprod/). MODIS Aqua is a moderate resolution satellite that images the Earth every 1–2 days, with data collected in 36 spectral bands. We use PAR data from the MODIS Level 3 8-day binned files. In order to calculate PAR anomaly, climatology data was used in order to establish a baseline. PAR variations are calculated using two different methods. The first is as an average of 12 satellite weeks of raw data. The second follows a method similar to the one for DHW, in which each weekly average is subtracted from the baseline maximum monthly PAR. In Table 1 the second measure is titled ‘PAR anomaly.’ As with DHW, a PAR anomaly value of 0 indicates no stress and increasingly positive numbers indicate increasing stress.

#### Dual formulations for DHW and PAR

For each of these stressors (DHW, PAR, and PAR anomaly), *observed* and *maximum* values are calculated. Observed DHW, for instance, represents the DHW calculated over the 12 weeks immediately preceding the observation (and therefore the stress *observed* up to the date of measurement). Maximum DHW represents the 12-week window between the January 1^st^, 2005 and the date of observation that records the highest DHW value. Maximum DHW, by construction, must be greater than or equal to observed DHW. We have used these two metrics to contrast continuous (or near-term) stress versus stress induced by past peak temperature or PAR events.

#### Population density

**Fig 2:**
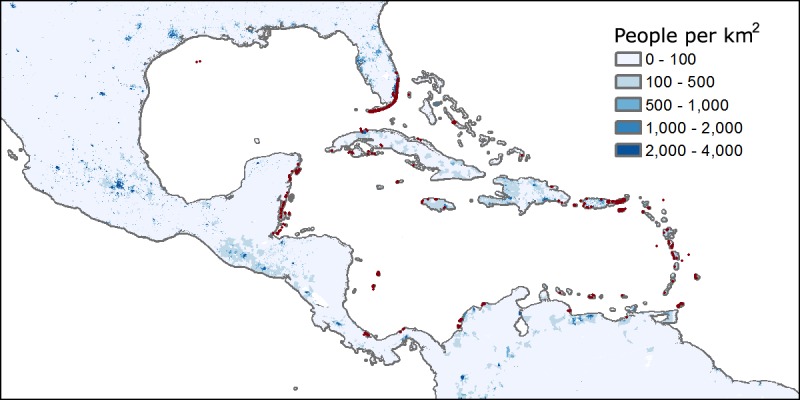
Population density in people per km^2^ (in blue) and coral observations (red dots). Figure produced by the authors using data from the GPWv4 dataset.

The maximum population density within a 50 kilometer radius from the site of coral observation was calculated using the UN-Adjusted Gridded Population of the World v4 (GPWv4) dataset estimates for 2005 (see Fig 2).

#### Distance from shore (DFS)

Distance from the reef location to shore was calculated using ArcGIS and is defined as the minimal distance from each coral observation to land as measured in kilometers.

#### Wind speed as a proxy for hurricane and storm intensity

Since 2005 was an active hurricane year in the Caribbean, it is important to control for possible mortality associated with storms. Maximum wind speed was used as a proxy for storm and hurricane stress. The parameter was calculated by finding the maximum wind speed that occurred between Jan 1, 2005 and the date of coral observation. Data was sourced from the NCEP-DOE Atmospheric Model Intercomparison Project (AMIP-II) reanalysis (R-2) project, and represents wind speed at 10 meters above sea level. AMIP-II is available at global scale, is reported twice-daily, and has a spatial resolution of 2.5 degrees.

Table 1 provides the summary statistics for the variables in the dataset. While a certain few locations report high mortality (maximum = 68%), the majority reported none at all (median = 0%; mean = 2%). In contrast, the median bleaching was nearly 26%. For temperature, it can be seen that almost half of the observations reported no temperature stress on the date of measurement (Observed DHW), and the typical location experienced what NOAA CRW would consider ‘mild bleaching’ at least once during the summer (Maximum DHW).

#### Controlling for spatial correlations

**Fig 3:**
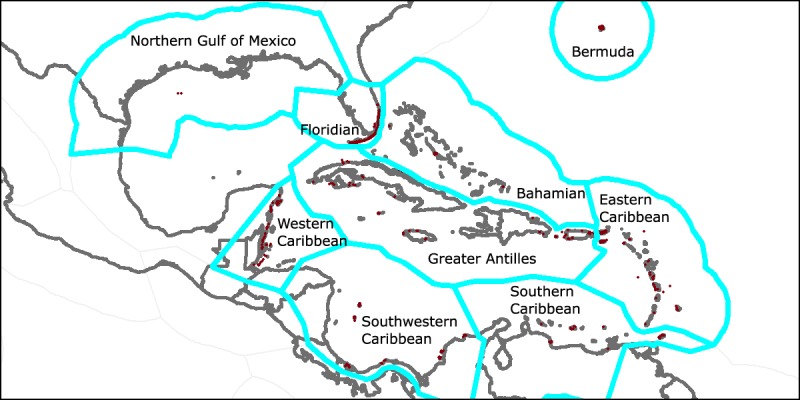
Marine Ecoregions of the World (MEOW) regions. Red points represent coral observations.

We control for spatial correlation between observations by introducing spatial fixed effects, using the eco-regional boundaries from the Marine Ecoregions of the World (MEOW). The data includes nine regions, as shown in Fig 3. The Northern Gulf of Mexico and Western Caribbean regions were lumped together since the Northern Gulf of Mexico region only contained 5 observations.

### Statistical modeling

As a part of the study, three major model specifications were tested–OLS, Tobit, and Fractional Logit. In S1 File we explain the assumptions that go with each of these modeling approaches. Here, and in the results, we present the results for the Fractional Logit model, since it performed best according to our selection criteria. While typical logit models are used to predict binary outcomes, by using a quasi-likelihood approach it is possible to repurpose the functional form so as to be able to use a continuous and bounded dependent variable [48]. It has been argued that Fractional Logit is more appropriate when the values outside the censored range are infeasible, and indeed this model best fits our results.

When considering spatial data, it is important to recognize that nearby observations will not be independent of one another. For example, due to larval dispersion in the water column, there is likely to be biological connectivity and similarity between nearby corals and reefs, as well as correlation between unobservable factors. To address this issue, we employ a fixed-effect regression that controls for spatial correlation. We include a spatial dummy for each of the nine ecoregions that span our data in the Caribbean as defined by the Marine Ecoregions of the World (MEOW) dataset. The fixed effects Fractional Logit model can be stated in a general form as follows, with *j* stressors (*x*) and *k* spatial dummies (*d*):

$$y_{i}=\frac{1}{1+e^{{-(\beta }_{0}+\sum_{j}\beta_{j}x_{ij}+\sum_{k}\beta_{k}d_{ik}+\epsilon_{i})}}$$ (1)

where *y*_*i*_ denotes that fraction of coral sampled at location *i* exhibiting mortality (Model 1) or bleaching (Model 2). As noted above, the dependent variables are calculated following Eakin et al. [43], as the average of the fraction of the area covered and the fraction of the colonies affected by mortality and bleaching, respectively. The exact model formulation was selected according to performance in a *k*-fold cross-validation. In *k*-fold cross-validation, the data is partitioned into *k* distinct groups, with each group acting as the test data set while the other *k -1* groups act to train the model. This process is repeated many times, and the reported model fit represents the average MSE across 1000 simulations.

## Results and discussion

The selected models for bleaching and mortality employ the same functional form as well as spatial controls (Eq 1), differing only in the included interaction terms and on whether average or maximum observed values are used in the independent variables. Mortality or bleaching, *y*, is predicted using a function of explanatory variables, *x*_*j*_, and spatial controls, *d*_*k*_. We find that different explanatory variables affect bleaching and mortality, and thus the variables used in the two models are highlighted in Table 1.

### Mortality

In Table 2 we show the estimated coefficients for mortality, as well as the marginal effects averaged across all data points in the sample. We find that maximum DHW, coral depth, and a coral depth-PAR Anomaly interaction term are all statistically significant in explaining coral mortality. Increases in maximum DHW lead to increased mortality, with an increase of 10 DHW leading to an approximate 4.9 percentage point increase in mortality. Similarly, an increase in PAR anomaly of 10 Einstein/m^2^·day would be associated with an increase in 0.8 percentage points in mortality, and an increase in 10 meters of the depth of a coral reef is associated with 9.7 percentage points less mortality. While population and wind speed are not statistically significant in the model, we find that their inclusion improved performance during cross-validation. Both parameters were positively correlated with mortality. Finally, we do find a significant difference in coral survival across regions, as seen in the regional marginal effects in Table 2. These effects can be interpreted as the change in expected mortality from a reference region, which has been set as the Western Caribbean / Northern Gulf of Mexico joint region. For example, according to the model the Eastern Caribbean region is expected to experience 4.7 percentage points less mortality than the reference given the same level of stress, while the Greater Antilles are expected to experience 4.9 percentage points more mortality. One region, Bermuda, shows itself to be an outlier. This indicates the corals in Bermuda showed themselves to be more robust than corals in other regions given the same level of stress, however it should be noted that this region reports the least data in the aggregate dataset with just 41 observations.

**Table 2:** Results for the regression model of coral mortality as a function of different explanatory variables.

*Variables*	*Coefficients*	*Average Marginal Effect*
Maximum DHW	0.304***	0.492***
	(0.050)	(0.071)
Maximum PAR Anomaly	-0.0073	0.0808***
	(0.015)	(0.018)
Population	0.0373	0.0679
	(0.031)	(0.057)
Wind	0.00114	0.00207
	(0.040)	(0.073)
Depth	-0.143***	-0.0969***
	(0.056)	(0.023)
Depth×Maximum PAR Anomaly	0.00517***	
	(0.0012)	
Depth×Maximum DHW	-0.00328	
	(0.0040)	
Bermuda	-10.9***	-93.1***
	(0.46)	(5.8)
Bahamian	1.56**	-6.2*
	(0.77)	(3.5)
Eastern Caribbean	0.474	-4.7***
	(0.35)	(1.6)
Greater Antilles	0.896**	4.9**
	(0.42)	(2.3)
Southern Caribbean	-2.08**	-7.4***
	(1.03)	(2.6)
Southwestern Caribbean	-0.380	-19.5***
	(0.35)	(2.2)
Floridian	-1.23***	-12.3***
	(0.47)	(2.2)
Constant	-5.74***	
	(0.89)	
*Log Likelihood* *Cross-validated MSE*	-64.34 30.64	
*Cross-validated RMSE*	5.53	
*Cross-validated R*^*2*^	0.325	
*Number of Observations*	1,045	

Estimated coefficients and average marginal effects using a fractional logit model, where coral mortality is explained as a function of environmental stressors. Robust standard errors are shown in parentheses (*** p<0.01, ** p<0.05, * p<0.1).

### Bleaching

In Table 3 we show the estimated coefficients for bleaching, as well as the marginal effects averaged across all data points in the sample. The marginal effect of temperature on bleaching is far larger than those of the other effects included in the model. Distance from shore, which is used as a proxy for coastal environmental stressors from human activities, is negatively associated with bleaching, indicating that, as expected, corals found further from land (and therefore stressors such as runoff or land use) are less likely to experience bleaching. Likewise, as with the mortality model, population density and wind speed were found to be positively associated with coral bleaching.

**Table 3:** Results for the regression model of coral bleaching as a function of different explanatory variables.

*Variables*	*Coefficients*	*Average Marginal Effect*
Observed DHW	0.201***	3.31***
	(0.014)	(0.16)
Maximum PAR Anomaly	0.0268***	-0.0274
	(0.0054)	(0.059)
Distance from Shore	-0.0363***	-0.72***
	(0.0137)	(0.27)
Population	0.0311***	0.618***
	(0.0059)	(0.12)
Wind	0.0132	0.263
	(0.0085)	(0.17)
Depth	0.0734***	0.375***
	(0.011)	(0.085)
Depth×Observed DHW	-0.00336**	
	(0.0010)	
Depth×Maximum PAR Anomaly	-0.00246***	
	(0.00049)	
Bermuda	0.114	-19.8***
	(0.137)	(1.7)
Bahamian	1.05***	2.8**
	(0.245)	(1.4)
Eastern Caribbean	0.0185	0.86
	(0.156)	(0.64)
Greater Antilles	0.325*	1.6**
	(0.188)	(0.77)
Southern Caribbean	1.16***	-3.8**
	(0.148)	(1.9)
Southwestern Caribbean	0.605***	-0.69
	(0.148)	(0.63)
Floridian	0.476**	-2.2**
	(0.176)	(0.89)
Constant	-2.421*** (0.198)	
Log-likelihood	-1299	
Cross-validated MSE	607.0	
Cross-validated RMSE	24.6	
Cross-validated R^2^	0.29	
Number of Observations	2,945	

Estimated coefficients and average marginal effects using a fractional logit model, where coral bleaching is explained as a function of environmental stressors. Robust standard errors are shown in parentheses (*** p<0.01, ** p<0.05, * p<0.1).

NOAA indicates that ‘mild bleaching’ can occur at DHW values larger than 4, and ‘severe bleaching’ at values of DHW larger than 8. The bleaching model suggests that an average DHW of 4 would be associated with a bleaching fraction of 37%, and an average DHW of 8 is associated with a bleaching of 53%. These high levels of bleaching may indicate an increasing sensitivity in the Caribbean to temperature. Lastly, PAR is found to be associated with increased bleaching, but only when phrased in terms of PAR anomaly, or radiation above baseline. This result indicates that corals adapt to their local microclimates with radiation as well as temperature.

Spatial variation across the Caribbean continues to significantly affect model results. Bermuda continues to report anomalous results, 19.8 percentage points below the reference region. The other regions report a 5.6 percentage point spread, the Southern Caribbean reporting the least bleaching tendencies and the Bahamian region the most. Accounting for separate spatial fixed effects is therefore crucial in maintaining unbiased estimates of the effects of stressors on coral health outcomes.

When assessing the final bleaching and mortality models, it is important to assess which elements have not been selected. For instance, while, as noted in the introduction, the literature suggests that a DHW–PAR interaction term has biological and laboratory support, it was not found to be superior in tests performed during the cross-validation. Likewise, in the bleaching model, maximum PAR Anomaly was selected over observed PAR anomaly, indicating that exposure to anomalous levels of radiation before the 12-week window is important in predicting current levels of bleaching.

**Fig 4:**
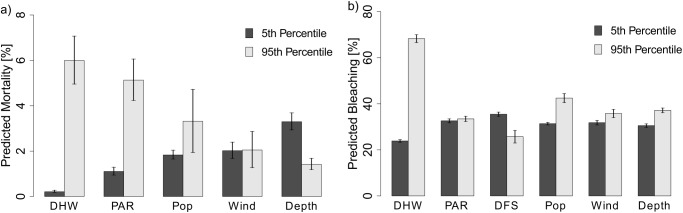
Relative effects of temperature (DHW), solar radiation (PAR), depth, and distance from shore (DFS). In Fig 4A, the predicted coral mortality is displayed while holding each stressor at the 5^th^ percentile and 95^th^ percentile of its observed values in the sample (while all other stressors are held at their mean). The error bars represent plus/minus two standard errors of the expected value. In Fig 4B the analogous information is reported using bleaching as the dependent variable.

To illustrate the sensitivity to the different stressors, in Fig 4 we show the difference between the predicted coral outcome when each of the stressors is varied between the 5^th^ and 95^th^ percentile of observed values in the sample (while all other variables are held at their means). Temperature is the major driver of both the bleaching and the mortality responses. Population density, the proxy for anthropogenic stress included in both models, plays a secondary effect in both models. For instance, in both the bleaching and mortality models the temperature effect is ~4 times larger than that for population density.

Hurricane stress, as measured by wind speed, plays a minor role in both models across the observed range. This tepid response is perhaps due to the cancelling out of the dual effects of hurricanes on coral health outcomes–hurricanes may increase stress on corals either directly or through increased runoff, or they may lessen stress due to upwelling [43,49].

In this study, we present further evidence of the effects from climatic and anthropogenic influences on coral reef ecosystem health. These effects are robust across a range of modeling strategies, and show that even after controlling for confounding mechanisms sea surface temperature plays a dominating role in controlling coral bleaching and mortality. Predictions indicate that in the Caribbean and across the world anomalous temperature events will rise in severity and become more frequent [11]. If, as estimated in Donner et al. [5], events of the magnitude of the 2005 Caribbean warming episode become bi-annual events in the next few decades, reef systems will degrade at accelerating rates.

Of note is that we find significant spatial differences between different geographic locales indicating varying ability of corals to respond to climate change. We also find interesting regional differences in correlations between stressors. For example, the correlation between distance from shore and depth varies across eco-regions, in some instances being high and positive, as is the case in the Southwestern Caribbean (ρ = 0.21), sometimes high and negative (for example, Bahamian, where ρ = -0.39), and in other instances a low correlation. More detail is included in S1 File. However, barring rapid and drastic biological acclimation, our results show that Caribbean reefs will face mounting existential pressure as the ocean continues to warm.

In conclusion, we find that that temperature and radiation far outweighed direct anthropogenic stressors in driving coral health outcomes during the 2005 Caribbean event. While selecting models based on performance using cross-validation decreases the risk of over-fitting, caution still must be used in generalizing the model to the modern Caribbean. Catastrophic events fundamentally change the nature of ecosystems, and it must be noted that the Caribbean of today cannot be completely represented by the Caribbean of 2005. However, the analysis presented in this paper provides additional evidence for the effect of temperature and other environmental stressors on coral health using a large sample of corals, and can be taken as suggestive of future trends both in the Caribbean and across the world. Indeed, the approach developed here can be used by other modelers and researchers to assess the effects for other locations, times and conditions, and it also provides a first order estimate that modelers can use in climate models to assess the effects of future environmental stressor states on coral health.

## Supporting information

S1 File Supplemental information. Discusses spatial distribution, multi-collinearity, the functional form of the regressions, the use of cross-validation for model choice, and within-region correlation patterns.
(PDF)
