# Supplementary material for: Estimating the effect of multiple environmental stressors on coral bleaching and mortality
Source: PLoS One. 2017 May 4;12(5):e0175018. doi: 10.1371/journal.pone.0175018 (PMC5417430; doi:10.1371/journal.pone.0175018)
Supplement: S1 File — Discusses spatial distribution, multi-collinearity, the functional form of the regressions, the use of cross-validation for model choice, and within-region correlation patterns. (PDF) [file pone.0175018.s001.pdf]

Supplemental Information

Estimating the effect of multiple environmental stressors on coral bleaching and mortality

Paul D. Welle, M.J. Small, Scott C. Doney, Inês L. Azevedo

SI1 Spatial Distribution 2
SI2 Addressing Multi-collinearity. 2
SI.3 Functional Forms for the Regressions 3
SI4 Using k-fold Cross-Validation to Identify the Best Model 4
SI5 Within-Region Correlation Tables 5

SI1 Spatial Distribution

Figure A shows the spatial distribution of the observations. The temporal distribution can be found in Figure 3 of the main text. There were three main concentrations of data: Belize/Mexico (~44%), The Florida Keys (~14%), and the eastern Caribbean (~25%).

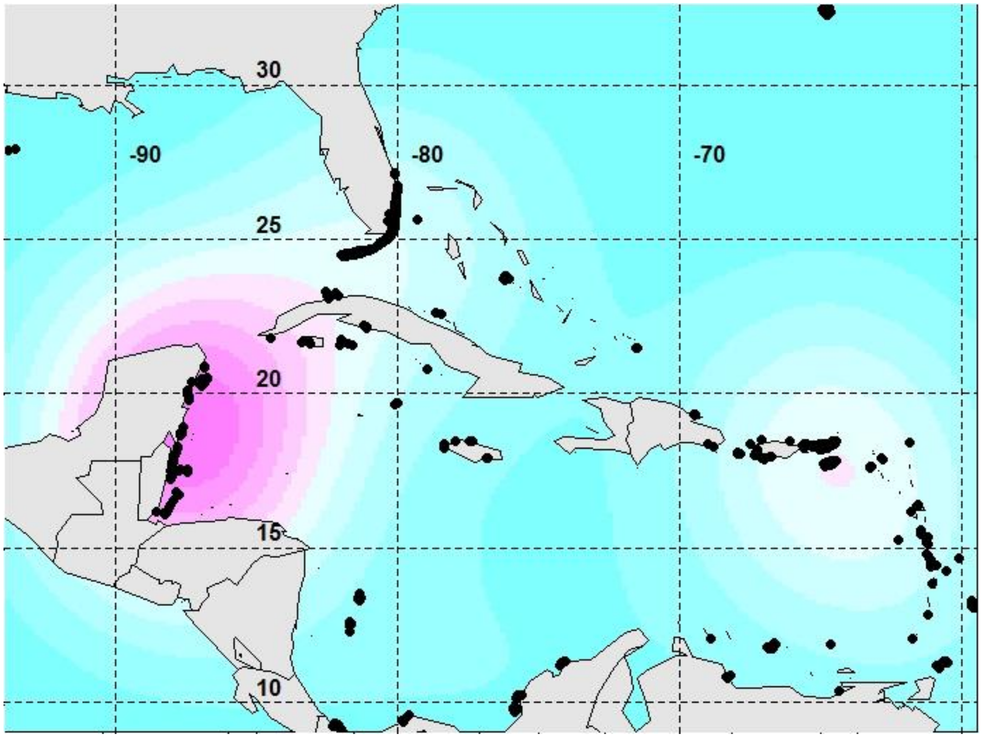

Figure A. Spatial distribution of observations. This figure shows spatial observations distributed over the Caribbean. Each black circle is an observation. The colors represent observation density, with pink showing high density and turquoise showing low.

SI2 Addressing Multi-collinearity.

When dealing with multiple measures in a regression analysis it is necessary to be careful to avoid constructing models with problems of multicollinearity, or high correlation between independent variables. Correlated independent variables result in large standard errors making statistical inference difficult. While multicollinearity does not bias the estimates directly, it can magnify biases caused by omitted variables or other causes. To avoid this, each of the models tested only includes one measure of each stressor (for instance maximum DHW or observed DHW, not both), with the presumption that different formulations of the same stressor will display high correlation. Table S1 shows the correlations between all variables, and indeed it can be seen that different formulations of the same stressor report correlation. Correlations across stressors (e.g. maximum DHW and maximum PAR anomaly) are generally low.

Table A. Correlations between stressors.
The upper-right triangle contains sample correlation coefficients.

	Maximum DHW	Observed DHW	Maximum PAR Anomaly	Observed PAR Anomaly	Distance From Shore	Population Density	Wind	Depth
**Maximum DHW**		0.67***	0.19***	0.11***	0.06***	-0.08***	-0.04*	0.15***
**Observed DHW**			-0.06***	-0.33***	0.03*	-0.09***	-0.07***	0.15***
**Maximum PAR Anomaly**				0.36***	-0.02	0.32***	0.06***	-0.10***
**Observed PAR Anomaly**					-0.11***	-0.01	-0.14***	0.00
**Distance From Shore**						-0.02	0.13***	0.10***
**Population Density**							0.05**	-0.09***
**Wind**								-0.13***
**Depth**								

P-values for statistical significance of indicated correlation coefficients:
*** p<0.01, ** p<0.05, * p<0.1

SI.3 Functional Forms for the Regressions

In Tables B and C, a series of models are presented along with measures of fit. The marginal effect of temperature is reported alongside to show the robustness of effect to model choice. For the purposes of these tables, two stressor forms are considered:

Univariate:

$$Bleaching_i = f(ObsDHW_i)$$

$$Mortality_i = f(MaxDHW_i)$$

Multivariate:

$$Bleaching_i = f(ObsDHW_i, ObsPAR\ Anomaly_i, Depth_i, DFS_i, PopDens_i, Wind_i, ObsDHW_i \times Depth_i, ObsPAR\ Anomaly_i \times Depth_i)$$

$$Mortality_i = f(MaxDHW_i, MaxPAR\ Anomaly_i, Depth_i, DFS_i, PopDens_i, Wind_i, MaxDHW_i \times Depth_i, MaxPAR\ Anomaly_i \times Depth_i)$$

The functional forms used are OLS, Tobit, and Fractional Logit. OLS, while being a common modeling strategy due to its simplicity, can have limitations under certain circumstances. It is not an appropriate choice for a censored dependent variable and will yield biased results. The model's linear formulation of independent variables can be misleading if the true data-generating process is not linear. Tobit allows for a censored dependent variable while maintaining the linear form of independent variables. Lastly, Fractional Logit would be more appropriate for modeling censored dependent variables with non-linear effects.

SI4 Using k-fold Cross-Validation to Identify the Best Model

We used a *k*-fold cross-validation for model selection. Cross-validation allows for simulation of out of sample prediction by withholding part of the data as a training set and uses part as testing set. In this study *k* was set equal to 5, meaning 80% of the data was iteratively used to predict the remaining 20%. Cross-validated R^2 and cross-validated MSE allow us to simulate which model is predicting best out of sample. The fractional logit model that controls for spatial effects performs best for both mortality and bleaching.

Table B: Mortality models and results.

Model	Description	Univariate /Multivariate	Spatial Control	Average Marginal Effect for Temperature	Cross-Validation R^2	Cross-Validation MSE
**OLS**	Standard linear regression	Univariate	None	0.660	0.15	38.59
**OLS**	Standard linear regression	Multivariate	None	0.666	0.20	36.32
**OLS**	Standard linear regression	Multivariate	Spatial Dummies	0.502	0.28	32.69
**Tobit**	Linear regression which accounts for censored dependent variable	Univariate	None	1.530	0.15	38.59
**Tobit**	Linear regression which accounts for censored dependent variable	Multivariate	None	1.680	0.18	37.23
**Tobit**	Linear regression which accounts for censored dependent variable	Multivariate	Spatial Dummies	1.861	0.08	41.77
**Fractional Logit**	Sigmoid which allows for non-linear effects	Univariate	None	0.594	0.17	37.68
**Fractional Logit**	Sigmoid which allows for non-linear effects	Multivariate	None	0.614	0.30	31.78
**Fractional Logit**	Sigmoid which allows for non-linear effects	Multivariate	Spatial Dummies	0.050	0.32	30.87

Table C. Bleaching models and results.

Model	Description	Univariate /Multivariate	Spatial Control	Average marginal effect for Temperature	Cross-Validation R^2	Cross-Validation MSE
**OLS**	Standard linear regression	Univariate	None	3.22	0.18	701.1
**OLS**	Standard linear regression	Multivariate	None	3.09	0.21	675.5
**OLS**	Standard linear regression	Multivariate	Spatial Dummies	3.28	0.25	641.3
**Tobit**	Linear regression which accounts for censored dependent variable	Univariate	None	3.54	0.18	701.1
**Tobit**	Linear regression which accounts for censored dependent variable	Multivariate	None	3.33	0.20	684.0
**Tobit**	Linear regression which accounts for censored dependent variable	Multivariate	Spatial Dummies	3.56	0.24	649.8
**Fractional Logit**	Sigmoid which allows for non-linear effects	Univariate	None	2.89	0.18	701.1
**Fractional Logit**	Sigmoid which allows for non-linear effects	Multivariate	None	2.70	0.23	658.4
**Fractional Logit**	Sigmoid which allows for non-linear effects	Multivariate	Spatial Dummies	2.99	0.27	624.2

SI5 Within-Region Correlation Tables

Table D. Correlations between stressors in the Reference Region - Joint Northern Gulf / Western Caribbean.

	Maximum DHW	Observed DHW	Maximum PAR Anomaly	Observed PAR Anomaly	Distance From Shore	Population Density	Wind	Depth
Maximum DHW		0.31***	-0.09	-0.09	-0.11	-0.02	0.08	0.04
Observed DHW			-0.11	-0.25***	0.06	-0.05	-0.09	0.01
Maximum PAR Anomaly				0.23***	-0.26***	0.73***	-0.73***	-0.10
Observed PAR Anomaly					-0.19***	0.34***	-0.22***	0.13
Distance From Shore						-0.37***	0.54***	0.03
Population Density							-0.68***	0.13
Wind								-0.15**
Depth								

P-values for statistical significance of indicated correlation coefficients: *** p<0.01, ** p<0.05, * p<0.1

Table E. Correlations between stressors in the Bermuda Region.

	Maximum DHW	Observed DHW	Maximum PAR Anomaly	Observed PAR Anomaly	Distance From Shore	Population Density	Wind	Depth
Maximum DHW		1.00***	-0.16	-0.33	-0.06	NA	NA	0.45**
Observed DHW			-0.16	-0.33	-0.06	NA	NA	0.45**
Maximum PAR Anomaly				0.91***	-0.38	NA	NA	-0.48**
Observed PAR Anomaly					-0.43**	NA	NA	-0.50**
Distance From Shore						NA	NA	-0.23
Population Density							NA	NA
Wind								NA
Depth								

P-values for statistical significance of indicated correlation coefficients:*** p<0.01, ** p<0.05, * p<0.1

Table F. Correlations between stressors in the Bahamian Region.

	Maximum DHW	Observed DHW	Maximum PAR Anomaly	Observed PAR Anomaly	Distance From Shore	Population Density	Wind	Depth
Maximum DHW		0.76***	-0.43***	-0.04	-0.03	0.95***	-0.49***	0.34**
Observed DHW			-0.31**	-0.62***	0.00	0.71***	-0.42**	0.26
Maximum PAR Anomaly				0.06	0.65***	-0.48***	0.20	-0.55***
Observed PAR Anomaly					0.06	-0.04	0.08	-0.09
Distance From Shore						-0.19	-0.06	-0.39**
Population Density							-0.48***	0.39**
Wind								-0.27
Depth								

P-values for statistical significance of indicated correlation coefficients: *** p<0.01, ** p<0.05, * p<0.1

Table G. Correlations between stressors in the Eastern Caribbean.

	Maximum DHW	Observed DHW	Maximum PAR Anomaly	Observed PAR Anomaly	Distance From Shore	Population Density	Wind	Depth
Maximum DHW		0.14***	0.14***	0.22***	-0.07	-0.10*	0.14***	-0.14***
Observed DHW			-0.56***	-0.71***	-0.06	-0.19***	-0.14***	0.11*
Maximum PAR Anomaly				0.59***	0.02	0.07	0.34***	-0.20***
Observed PAR Anomaly					-0.06	0.03	0.03	-0.14***
Distance From Shore						-0.21***	0.29***	0.13***
Population Density							0.01	-0.01
Wind								-0.06
Depth								

P-values for statistical significance of indicated correlation coefficients: *** $p<0.01$, ** $p<0.05$, * $p<0.1$

Table H. Correlations between stressors in the Greater Antilles.

	Maximum DHW	Observed DHW	Maximum PAR Anomaly	Observed PAR Anomaly	Distance From Shore	Population Density	Wind	Depth
Maximum DHW		0.83***	0.01	-0.03	-0.11	0.09	-0.22	0.22
Observed DHW			-0.05	-0.30**	-0.09	0.22	-0.27**	0.09
Maximum PAR Anomaly				0.23	0.03	0.12	0.23	-0.05
Observed PAR Anomaly					0.01	0.05	-0.30**	-0.01
Distance From Shore						0.30**	0.22	-0.08
Population Density							-0.08	-0.11
Wind								0.07
Depth								

P-values for statistical significance of indicated correlation coefficients: *** $p<0.01$, ** $p<0.05$, * $p<0.1$

Table I. Correlations between stressors in the Southern Caribbean.

	Maximum DHW	Observed DHW	Maximum PAR Anomaly	Observed PAR Anomaly	Distance From Shore	Population Density	Wind	Depth
Maximum DHW		0.97***	0.17	-0.54***	-0.11	-0.06	0.83***	0.09
Observed DHW			0.20	-0.54***	-0.09	-0.11	0.77***	0.06
Maximum PAR Anomaly				0.21	0.19	0.08	-0.01	0.00
Observed PAR Anomaly					-0.06	0.41***	-0.64***	-0.07
Distance From Shore						-0.19	0.07	0.01
Population Density							0.07	-0.13
Wind								0.09
Depth								

P-values for statistical significance of indicated correlation coefficients: *** $p<0.01$, ** $p<0.05$, * $p<0.1$

Table J. Correlations between stressors in the Southwestern Caribbean.

	Maximum DHW	Observed DHW	Maximum PAR Anomaly	Observed PAR Anomaly	Distance From Shore	Population Density	Wind	Depth
Maximum DHW		0.31^{***}	-0.01	0.30^{***}	0.00	0.41^{***}	-0.41^{***}	-0.19
Observed DHW			-0.38^{***}	-0.07	0.00	0.18	-0.51^{***}	-0.19
Maximum PAR Anomaly				0.51^{***}	-0.27^{***}	-0.42^{***}	-0.03	-0.17
Observed PAR Anomaly					-0.10	-0.26^{***}	-0.26^{***}	-0.17
Distance From Shore						-0.01	0.07	0.21^{*}
Population Density							0.14	0.16
Wind								0.44^{***}
Depth								

P-values for statistical significance of indicated correlation coefficients: *** $p<0.01$, ** $p<0.05$, * $p<0.1$

Table K. Correlations between stressors in the Floridian region.

	Maximum DHW	Observed DHW	Maximum PAR Anomaly	Observed PAR Anomaly	Distance From Shore	Population Density	Wind	Depth
Maximum DHW		0.31^{***}	-0.09	-0.09	-0.11	-0.02	0.08	0.04
Observed DHW			-0.11	-0.25^{***}	0.06	-0.05	-0.09	0.01
Maximum PAR Anomaly				0.23^{***}	-0.26^{***}	0.73^{***}	-0.73^{***}	-0.10
Observed PAR Anomaly					-0.19	0.34^{***}	-0.22^{***}	0.13
Distance From Shore						-0.37^{***}	0.54^{***}	0.03
Population Density							-0.68^{***}	0.13
Wind								-0.15^{**}
Depth								

P-values for statistical significance of indicated correlation coefficients: *** $p<0.01$, ** $p<0.05$, * $p<0.1$
